# Tolerance mechanisms in polysaccharide biosynthesis: Implications for undecaprenol phosphate recycling in *Escherichia coli* and *Shigella flexneri*

**DOI:** 10.1371/journal.pgen.1011591

**Published:** 2025-01-30

**Authors:** Jilong Qin, Yaoqin Hong, Nicholas T. Maczuga, Renato Morona, Makrina Totsika

**Affiliations:** 1 Centre for Immunology and Infection Control, School of Biomedical Sciences, Queensland University of Technology, Brisbane, QLD, Australia; 2 Max Planck Queensland Centre, Queensland University of Technology, Queensland, Australia; 3 School of Biological Sciences, Department of Molecular & Biomedical Sciences, Research Centre for Infectious Diseases, University of Adelaide, Adelaide, Australia; University of Wisconsin-Madison, UNITED STATES OF AMERICA

## Abstract

Bacterial polysaccharide synthesis is catalysed on the universal lipid carrier, undecaprenol phosphate (UndP). The cellular UndP pool is shared by different polysaccharide synthesis pathways including peptidoglycan biogenesis. Disruptions in cytosolic polysaccharide synthesis steps are detrimental to bacterial survival due to effects on UndP recycling. In contrast, bacteria can survive disruptions in the periplasmic steps, suggesting a tolerance mechanism to mitigate UndP sequestration. Here we investigated tolerance mechanisms to disruptions of polymerases that are involved in UndP-releasing steps in two related polysaccharide synthesis pathways: that for enterobacterial common antigen (ECA) and that for O antigen (OAg), in *Escherichia coli* and *Shigella flexneri*. Our study reveals that polysaccharide polymerisation is crucial for efficient UndP recycling. In *E*. *coli* K-12, cell survival upon disruptions in OAg polymerase is dependent on a functional ECA synthesis pathway and vice versa. This is because disruptions in OAg synthesis lead to the redirection of the shared lipid-linked sugar substrate UndPP-GlcNAc towards increased ECA production. Conversely, in *S*. *flexneri*, the OAg polymerase is essential due to its limited ECA production, which inadequately redirects UndP flow to support cell survival. We propose a model whereby sharing the initial sugar intermediate UndPP-GlcNAc between the ECA and OAg synthesis pathways allows UndP to be redirected towards ECA production, mitigating sequestration issues caused by disruptions in the OAg pathway. These findings suggest an evolutionary buffering mechanism that enhances bacterial survival when UndP sequestration occurs due to stalled polysaccharide biosynthesis, which may allow polysaccharide diversity in the species to increase over time.

## Author summary

Enzymes involved in bacterial polysaccharide biosynthesis have substrate specificity to ensure the correct polysaccharide is produced at the appropriate place and time. However, this specificity poses a challenge for the diversification of polysaccharide structure and hence function, as the acquisition of a novel oligosaccharide repeating unit would likely disrupt the synthesis pathways, leading to sequestration of the essential universal lipid carrier UndP and ultimately cause cell death. We investigated how cells tolerate disruptions in polysaccharide synthesis pathways and provide evidence that suggests that sharing a common substrate between the synthesis pathways of two common enteric bacterial surface polysaccharides (ECA and OAg), can redirect the flow of UndP. Our study provides insights into the mechanism of how bacteria alleviate sequestration issues, thereby enhancing cell survival which may allow them additional capacity for polysaccharide diversification.

## Introduction

Surface polysaccharides confer enteric bacteria with resistance to host antimicrobials [1,2] and the ability to colonise various host niches [3]. The O antigen (OAg) polysaccharide attached to lipid A–core oligosaccharide of lipopolysaccharide molecules is made of oligosaccharide repeating units (RUs) and represents an important virulence factor for Gram-negative bacteria contributing to host colonisation [4] and virulence modulation [5]. The structure of OAg is under strong selection by host immunity and host niche-residing bacteriophages, giving rise to over 180 diverse OAg structures characterised to date in *Escherichia coli* (including *Shigella* strains) [6], which form the molecular basis of the O-typing scheme [7].

**Fig 1:**
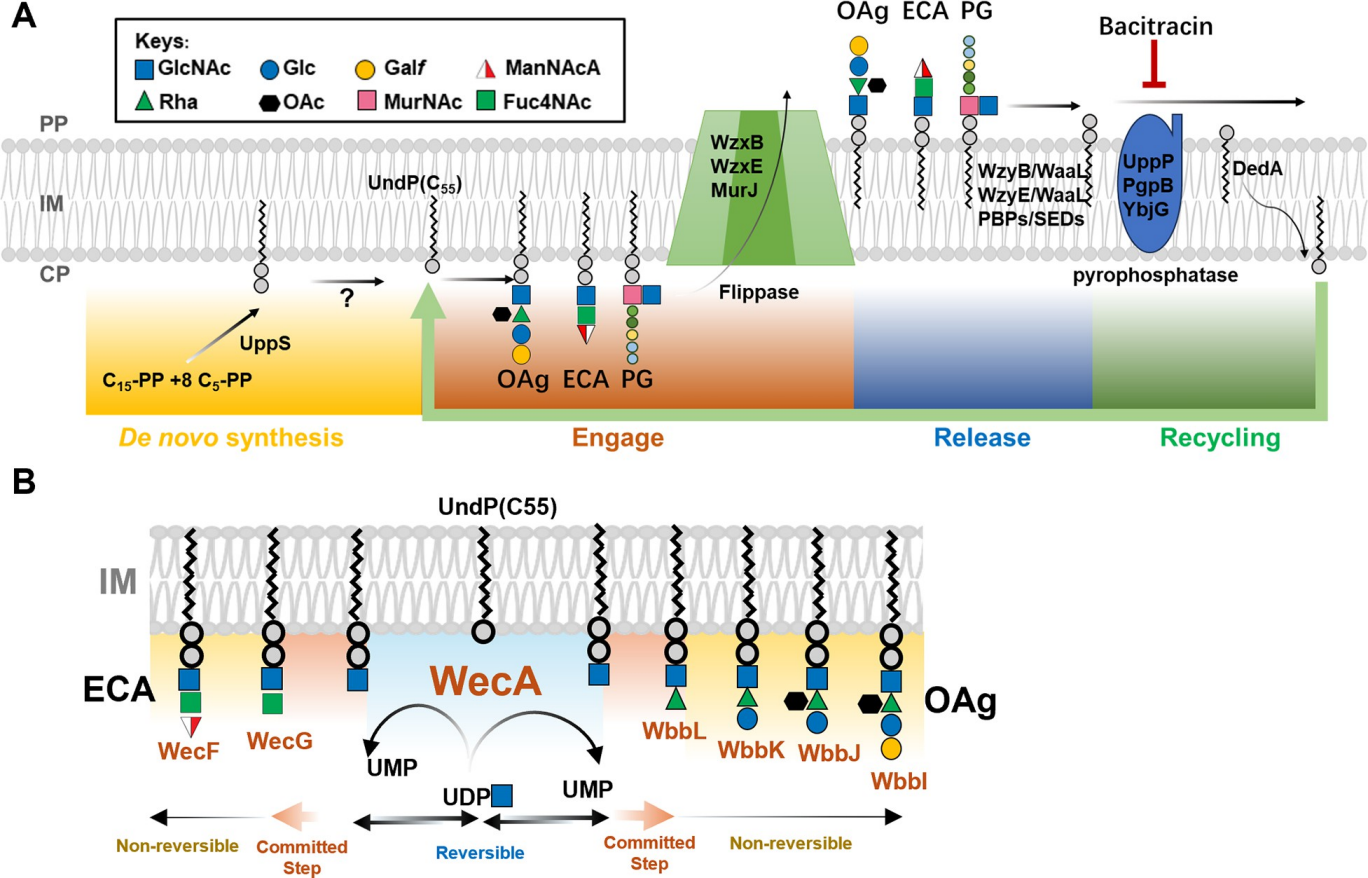
Biogenesis of bacterial polysaccharides on the universal lipid carrier UndP. A) Schematic representation of UndP *de novo* synthesis, glycan RU engagement, release and recycling during OAg, ECA and peptidoglycan biosynthesis in *E*. *coli*. PP, periplasm; IM inner membrane; CP, cytoplasm. B) Schematic representation of the biogenesis of ECA and OAg RUs on the UndP lipid carrier with the shared initial saccharide GlcNAc. Glycosyltransferases responsible for saccharide assembly are shown in orange.

The biosynthesis of OAg relies on the universal lipid carrier undecaprenol phosphate (UndP or C_55_-P), which is shared with the biosynthesis of other polysaccharides, such as enterobacterial common antigen (ECA) and peptidoglycan in the cell (Fig 1A). However, cells only produce a finite amount of UndP (<1% of total membrane lipids [8]) through *de novo* synthesis (Fig 1A), and the demand for UndP during synthesis of different polysaccharides is critically ensured through efficient recycling (Fig 1A). In the biosynthesis cycle of OAg and ECA for most *E*. *coli* and *S*. *flexneri* strains, UndP is engaged by the initial transferase, *N*-acetylglucosamine-1-phosphate transferase WecA, to form UndPP-GlcNAc in an enzymatically reversible manner [9] (Fig 1B). UndP is then committed to either the OAg or ECA synthesis cycle in the subsequent step catalysed by the second glycosyltransferase, WbbL (for OAg in *E*. *coli* K-12 [10]) or WecG (for ECA), and remains occupied by RUs during their assembly (Fig 1B), including the subsequent membrane-translocation step by flippases WzxB and WzxE, respectively (Fig 1A). UndPP is then released in the periplasm from UndPP-OAg (by OAg polymerase WzyB and ligase WaaL in a competitive manner [11]), and UndPP-ECA (by ECA polymerase WzyE and ligase WaaL) (Fig 1A). While both released ECA and OAg RUs can be ligated onto LPS by WaaL to form ECA_LPS_ and smooth LPS (S-LPS), respectively, ECA can also be ligated to phospholipids to generate phosphatidylglycerol-linked ECA (ECA_PG_) on the bacterial cell surface, or resides in the periplasm in a cyclic form (ECA_cyc_), through a process that remains to be defined [12]. For peptidoglycan synthesis, UndP is engaged after RU assembly by MraY, translocated by MurJ, and released by penicillin-binding proteins (PBPs) and proteins belonging to the SEDS (shape, elongation, division, and sporulation) family (Fig 1A). The released UndPP is further dephosphorylated into UndP by pyrophosphatases and translocated back into the cytosolic leaflet for subsequent rounds of synthesis (Fig 1A).

The enzymes collectively responsible for the biosynthesis and assembly of different polysaccharides recognise the substrates with a common feature, UndPP-linked glycans. To ensure that the different polysaccharides are synthesised and assembled correctly, synthesis pathways have specificities towards glycan moieties on the lipid carrier in three different processes: step-wised assembly of UndPP-RUs through glycosyltransferases, IM translocation of UndPP-RUs through flippases, and polymerisation of RUs through polymerases. The specificity of glycosyltransferases is also towards acceptors (UndPP-glycans), and disruptions in glycosyltransferases will abolish or stall the RU assembly on UndPP [13]. Flippase specificity ensures that only the complete UndPP-RUs are efficiently translocated across the IM, leaving any incomplete RUs in the cytoplasmic leaflet of the IM [14]. Polymerases (Wzy) only efficiently polymerise the specific RUs for a given pathway [15]. Therefore, genes coding for OAg glycosyltransferases, flippases and polymerases have high sequence diversity and are often specific to an individual OAg gene cluster, a feature that has been exploited as a molecular serotyping method for rapid strain identification and clinical detection [16].

Although enzyme specificities are crucial to the quality control of polysaccharide biosynthesis, they may represent a limitation in the evolutionary diversification process for their polysaccharide RU structures for serotype switching. We have previously shown that expression of the mono-rhamnosyltransferase WbbL in *S*. *flexneri* that is defective in di-rhamnosyltransferase RfbG resulted in cell lysis [13]. This is because the incomplete OAg RU intermediate UndPP-GlcNAc-Rha catalysed by WbbL is not recognised neither by the last glycosyltransferase RfbF nor the flippase WzxB in *S*. *flexneri*, causing sequestration of UndP in OAg biosynthesis, thereby limiting the availability of UndP for peptidoglycan synthesis. This finding is consistent with previous studies [17,18], which suggest that cell shape abnormalities resulting from defects in peptidoglycan synthesis—caused by disruptions in the late steps of OAg biogenesis—are due to a limited supply of UndP. These abnormalities can be alleviated by overexpressing UppS, which increases the *de novo* synthesis of UndPP. Consequently, direct genetic disruptions in the late steps of polysaccharide biogenesis, including ECA, OAg, and capsule polysaccharide [19–21], may be difficult to achieve due to potential lethal stress, which can select for secondary suppressor mutations. These observations suggested that the evolution of a new OAg RU type (serotype switching) comes with a risk of deleterious effects, in that the newly incorporated functional glycosyltransferase may stall the existing OAg synthesis due to specificities of polysaccharide enzymes, leading to UndP sequestration and cell death. Therefore, understanding bacterial tolerance to UndP sequestration, particularly in the context of disruptions to synthesis pathways, can provide insights into the evolution of polysaccharide diversification, a widespread biological process in bacteria.

Herein, we studied the survival and tolerance of *E*. *coli* and *S*. *flexneri* to UndP sequestration in mutants defective in UndPP-releasing steps during both OAg and ECA synthesis. We showed that *E*. *coli* and *S*. *flexneri* have different tolerance to the disruption of OAg polymerases. The high tolerance to OAg polymerase disruption in *E*. *coli* was due to increased redirection of the substrate UndPP-GlcNAc towards ECA synthesis, whereas the low tolerance to OAg polymerase disruption in *S*. *flexneri* was due to the low ECA synthesis capacity of this bacterium. Our data suggest a buffering mechanism, where through sharing the initial substrate UndPP-GlcNAc between OAg and ECA synthesis, *E*. *coli* can alleviate UndP sequestration stress during OAg synthesis by redirecting UndP flow to the ECA synthesis cycle and ensure increased cell survival.

## Results

### The OAg polymerase WzyB contributes to rapid UndP recycling

**Fig 2:**
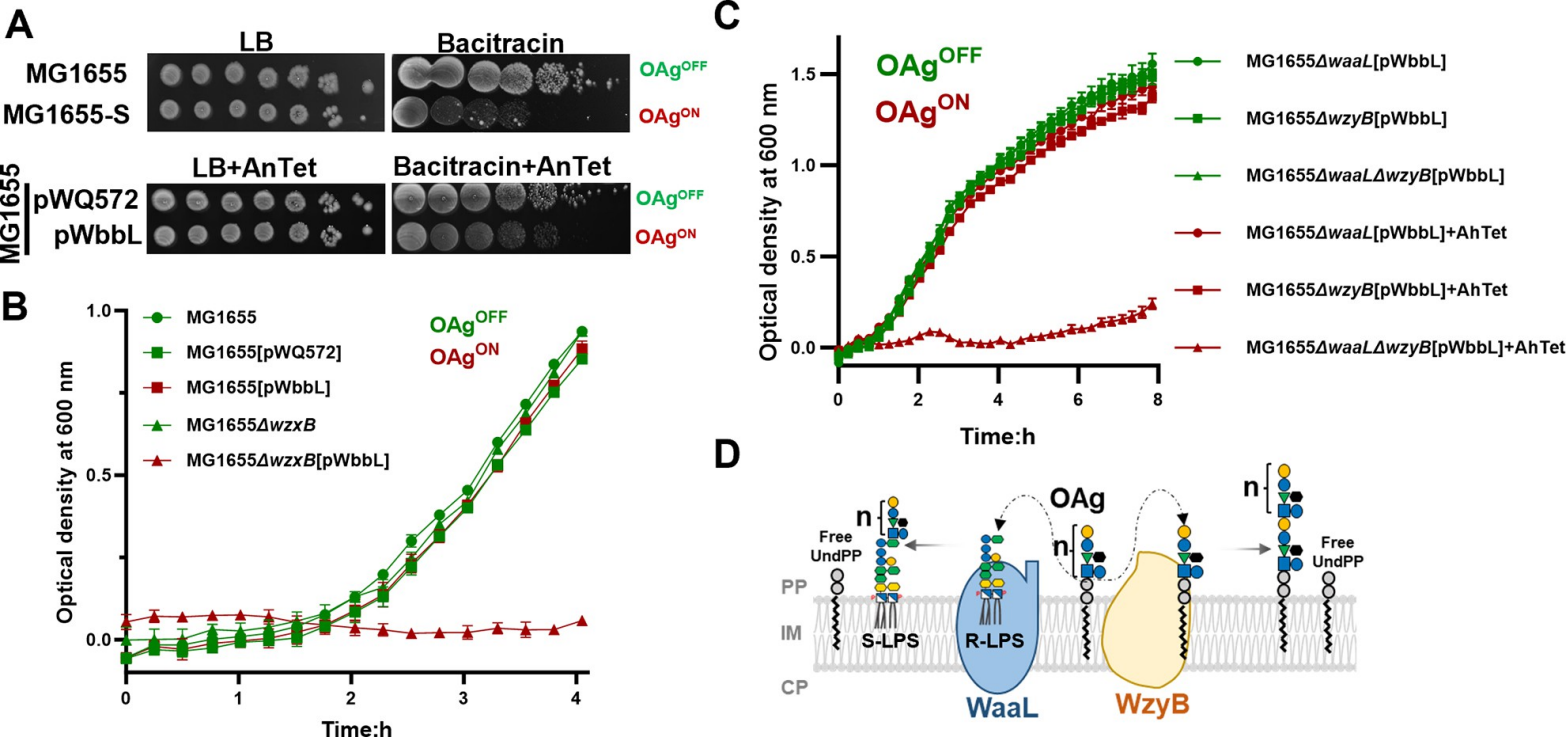
Synthetic lethality of *E*. *coli* Δ*waaL*Δ*wzyB* mutant due to complete stalling of UndPP-OAg intermediates. **A)** Bacitracin sensitivity assay for *E*. *coli* MG1655 and its derivative strains. Overnight bacterial cultures were 10-folded diluted and spotted on LB agar plates supplemented without or with 1 mg/ml bacitracin. **B-C)** Growth curves of *E*. *coli* K-12 strains harbouring plasmids without or with *wbbL* cultured in LB media supplemented with or without anhydrotetracycline (AnTet). The status of OAg production is labelled as OAg^ON^ in red, or OAg^OFF^ in green. **D)** Schematic representation of enzymatic reactions catalysed by WaaL and WzyB in releasing UndPP from UndPP-OAg RUs. n = 17–21 in *E*. *coli* K-12 for O16-OAg.

Biosynthesis of OAg occupies a pool of UndP [22]. In *E*. *coli* K-12 strain MG1655, OAg synthesis is inactive due to the disruption of *wbbL* gene encoding the glycosyltransferase responsible for the committed step of OAg RU assembly (Fig 1B). Consequently, expression of WbbL in MG1655, or restoration of OAg synthesis in *E*. *coli* K-12 strain MG1655-S by removal of IS element in *wbbL* gene [23] increased sensitivity towards bacitracin, an antibiotic targeting the pyrophosphatases for UndP recycling (Fig 1A), without affecting cell viability (Fig 2A). Limiting the level of UndP by sequestering it in OAg or ECA synthesis has a negative impact on cell survival as it limits the synthesis of essential cell wall component peptidoglycan. In *E*. *coli* K-12 strain MG1655, switching on OAg production by inducing the committed-step glyosyltransferase WbbL in a flippase *wzxB* mutant background inhibits cell growth (Fig 2B). Disruption of the UndPP-OAg flippase *wzxB* has been shown to sequester UndP in UndPP-OAg intermediates by radioactive labelling [22], and disruptions in late glycosyltransferases WbbK and WbbJ, which also sequester UndP in incomplete UndPP-OAg intermediates were documented with cell shape deformities [18], a hallmark of peptidoglycan synthesis defects. The cell shape abnormalities in the late glycosyltransferase mutants were shown to be fully rescued by overexpression of UppS (to increase UndPP *de novo* synthesis) or MurA (to increase competition for UndP in peptidoglycan synthesis), suggesting that the cell death was due to UndP shortage in peptidoglycan synthesis pathways [18]. These results collectively confirmed that sequestration of UndP in the UndPP-linked OAg RU intermediates in the cytosolic face of IM is lethal. In contrast, in the WbbL-complemented MG1655 strain background (Fig 2C), disruption of the OAg ligase WaaL, known to sequester UndP in the UndPP-OAg intermediates in the periplasmic leaflet [22,23], is not lethal. We reasoned that the polymerisation reaction by WzyB in the absence of WaaL releases enough UndPP to be recycled for peptidoglycan synthesis (Fig 2D), and therefore only the disruption of both WaaL and WzyB would be lethal. Indeed, while the absence of either WaaL or WzyB had no impact on cell growth, lack of both WzyB and WaaL inhibited cell growth (Fig 2C). These results suggested that the OAg polymerase WzyB contributes to rapid UndP recycling. Our findings are in line with a previous report showing that disruption of *wzyB* accumulated a high level of UndPP-OAg RUs [22].

### Disruption of wzyB in S. flexneri is lethal

**Fig 3:**
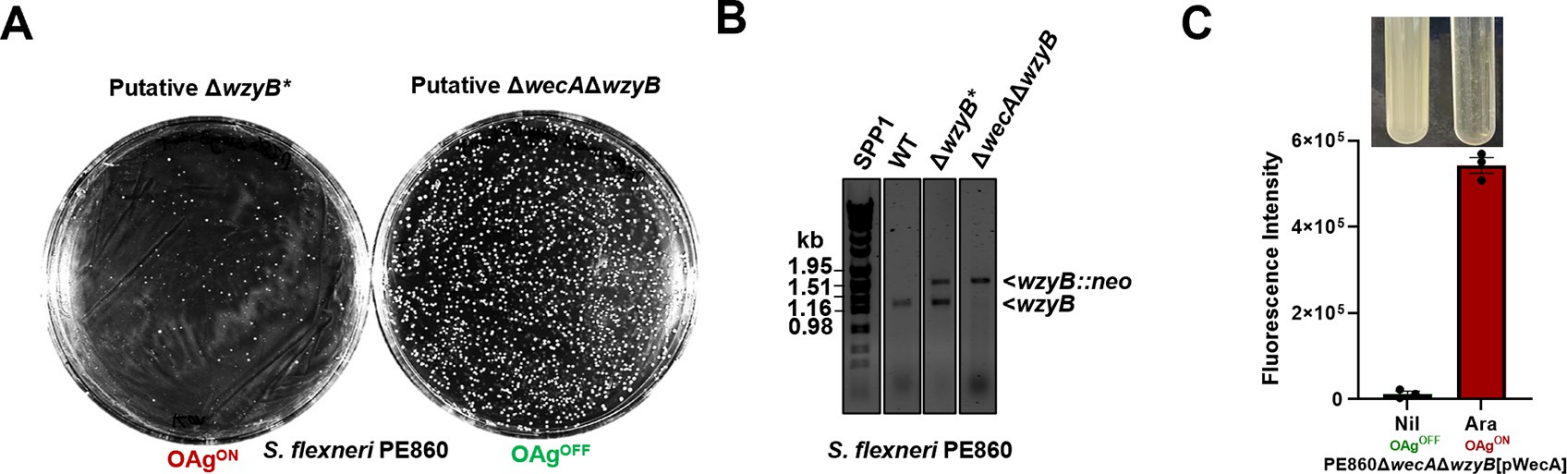
WzyB is essential in *S*. *flexneri*. **A)** Colony number and morphology of putative *wzyB* inactivation mutants in either the background of WT or *wecA*-null *S*. *flexneri*. **B)** *wzyB*-specific PCR for *wzyB* inactivation with primers targeting coding sequences of WzyB. Successful insertions of *neo* in *wzyB* is indicated as *wzyB*::*neo*. **C)** Amount of cellular DNA detected by ethidium bromide (EtBr) in *S*. *flexneri* Δ*wecA*Δ*wzyB* culture supernatant released upon pWecA induction. Cell lysis was imaged as loss of turbidity in culture media. The status of OAg production is labelled as either OAg^ON^ in Red or OAg^OFF^ in Green.

Interestingly, gene encoding OAg polymerase WzyB in *S*. *flexneri*, together with a previously confirmed essential gene *rfbF* [13] were reported to completely lack transposon insertions in a dense transposon insertion library, unlike genes up- and down-stream (*rfbBDAC* and *rfbJ*, respectively) [24], strongly indicating that WzyB is essential in *S*. *flexneri*. Here, we found that attempting to construct a direct *wzyB* deletion in *S*. *flexneri* resulted in a few small and slow-growing colonies (Fig 3A). By a *wzyB*-specific PCR, it was confirmed that the putative *wzyB* mutant colonies contained a *wzyB* duplication, which likely acts as a suppressor mutation (Fig 3B). Since WzyB contributes to rapid UndP recycling, we suspected that disruption of *wzyB* may cause UndP sequestration in *S*. *flexneri*, impacting its growth. We therefore made the *wzyB* deletion in an initial transferase Δ*wecA* mutant background (which is disrupted for UndP engagement in both ECA and OAg synthesis) to avoid potential UndP sequestration and confirmed this to be the case by successful deletion of *wzyB* in this background (Fig 3A–3B). Strikingly, complementation of WecA expression in the Δ*wecA*Δ*wzyB* double mutant resulted in cell lysis with the release of cellular DNA into culture supernatant (Fig 3C). These results suggest that WzyB is essential in *S*. *flexneri*, implying that the deletion of *wzyB* causes UndP sequestration (via OAg RU buildup) leading to cell death.

### Redirection of UndPP-GlcNAc between ECA and OAg to mitigate UndP sequestration

**Fig 4:**
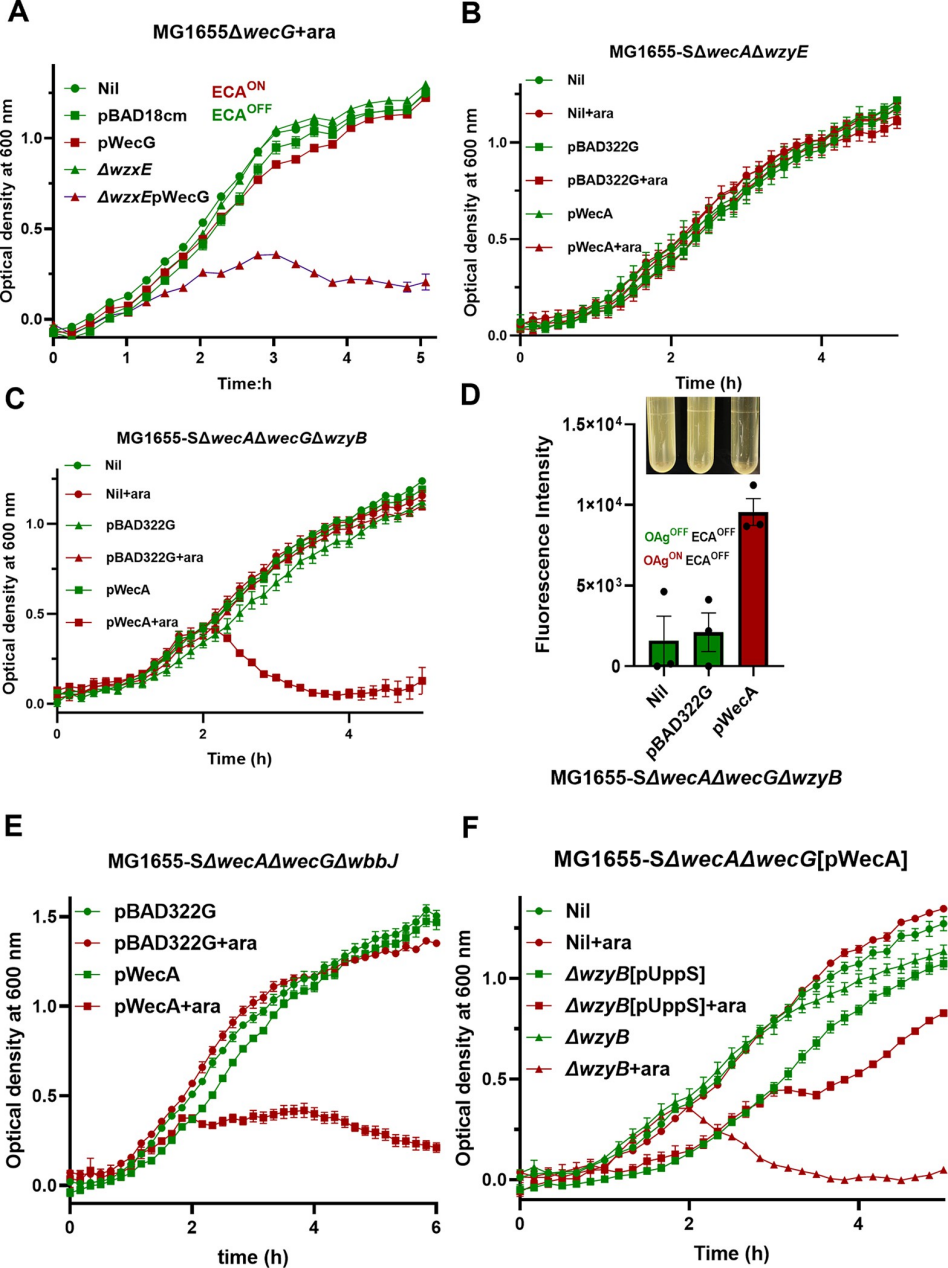
Interdependence of *wzy* essentiality between OAg and ECA synthesis pathways. **A-C)** Growth curves of indicated *E*. *coli* K-12 strains harbouring plasmids either without or with *wecG* (**A**) or *wecA* (**B, C, E &F**) cultured in LB media supplemented either with or without arabinose (ara). **D)** Release of cellular DNA of MG1655-SΔ*wecA*Δ*wecG*Δ*wzyB* in culture supernatant upon pWecA induction detected by ethidium bromide (EtBr). Cell lysis was imaged as loss of turbidity in culture media. The status of OAg production is labelled as either OAg^ON^ in Red or OAg^OFF^ in Green.

Besides the OAg polymerase WzyB being essential in *S*. *flexneri*, the ECA polymerase WzyE was also found to be essential in *E*. *coli* K-12 strains lacking OAg due to inactivation of the gene encoding for the committed-step glycosyltransferase WbbL. This is potentially due to the sequestration of UndP in the UndPP-ECA intermediates, since we have generated evidence suggesting that polymerase Wzy is crucial for a rapid UndPP recycling. Indeed, accumulation of dead-end intermediates in the ECA pathway were shown to induce cell morphological defects through UndP sequestration, adversely affecting peptidoglycan synthesis [17]. In addition, accumulation of UndPP-ECA intermediates in strains with disruptions of *wzxE* was shown previously to be lethal [25]. We also showed here that the induction of WecG in the MG1655 Δ*wecG*Δ*wzxE* double mutant lacking OAg resulted in cell death (Fig 4A), confirming that the sequestration of UndP in ECA synthesis route in *E*. *coli* K-12 is also lethal. Interestingly, *wzyE* was not essential in *E*. *coli* strains producing OAg (ST131) [26]. Moreover, *wzyE* could be deleted in *E*. *coli* K-12 strain MG1655-S restored for OAg production (Table 1). We also successfully deleted *wzyE* in MG1655-SΔ*wecA* (disruption in engaging UndP for both OAg and ECA synthesis). In this strain, switching on both ECA and OAg production by inducing WecA in a Δ*wecA*Δ*wzyE* double mutant revealed no growth defects, suggesting that when OAg is produced, WzyE is not essential (Fig 4B). We reasoned that this is because, in most *E*. *coli* strains, OAg shares the same first sugar *N*-acetylglucosamine (GlcNAc) with ECA, allowing the common substrate UndPP-GlcNAc to be redirected into making OAg (Fig 1B) when UndP is sequestered in a *wzyE* mutant. This prompted us to examine the essentiality of the OAg polymerase WzyB in the OAg-restored *E*. *coli* K-12 strain with ECA synthesis inactivated (MG1655-SΔ*wecA*Δ*wecG*Δ*wzyB*). Intriguingly, when ECA synthesis is inactivated through the disruption of committed step catalysed by WecG (Fig 1B), induction of WecA in MG1655-SΔ*wecA*Δ*wecG*Δ*wzyB* resulted in cell lysis (Fig 4C–4D), suggesting that the OAg polymerase WzyB is essential when ECA synthesis is inactivated. Disruptions of genes responsible for late glycosyltransferases *wbbK* and *wbbJ* and flippase *wzxB* in an OAg producing MG1655 strain background were well characterised previously with cell morphological changes due to sequestration of UndP in the UndPP-linked OAg RU intermediates [18], and the cell abnormalities in these mutants were further dampened when ECA biosynthesis was inactivated. However, the growth kinetics for these mutant strains were not previously characterised. To confirm that sequestration of UndPP-linked incomplete OAg RU intermediates in MG1655-S is lethal when ECA is inactivated, we generated the triple mutant MG1655-SΔ*wecA*Δ*wecG*Δ*wbbJ* and confirmed that the induction of WecA in this mutant causes stalled cell growth followed by cell lysis indicated by a decrease in cell culture turbidity (Fig 4E). Therefore, lethality of MG1655-SΔ*wecG*Δ*wzyB* (Fig 4C) is also likely to be due to the sequestration of UndP in UndPP-linked OAg intermediates. Indeed, increasing the *de novo* synthesis of UndPP by overexpression of UppS rescued the lethality of MG1655-SΔ*wecG*Δ*wzyB* (Fig 4F). These results strongly suggests that cell lethality observed in strains with disruptions in late OAg synthesis steps were due to sequestration of UndP. Together, these results led us to propose a model that by sharing a common initiating sugar GlcNAc between ECA and OAg, bacteria could gain increased tolerance to UndP sequestration through redirecting their common substrate UndPP-GlcNAc into remaining functional synthesis pathways to ensure rapid UndP recycling and cell viability.

### Low tolerance to UndP sequestration in S. flexneri due to limited ECA production

**Fig 5:**
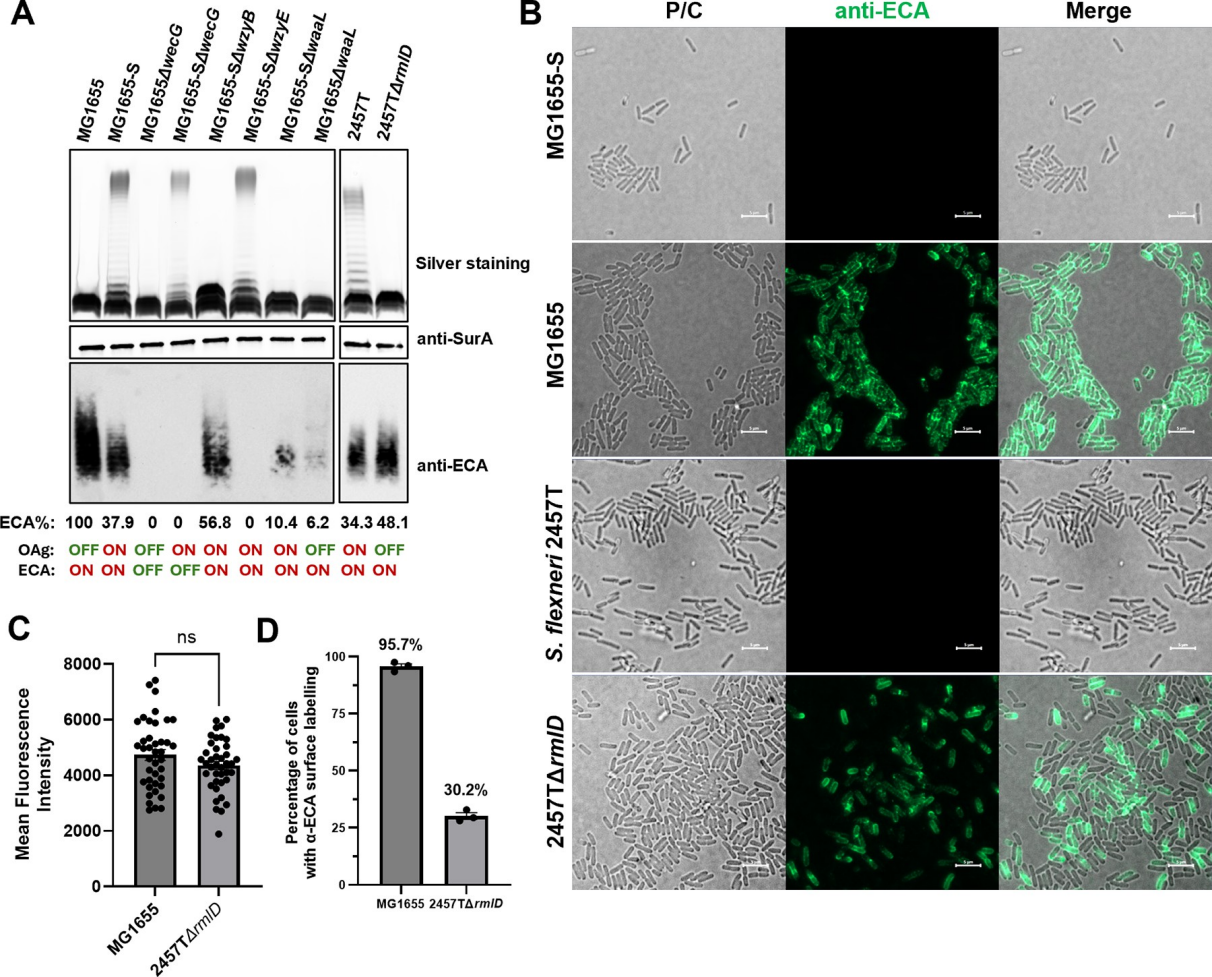
Different ECA biosynthesis levels revealed between *E*. *coli* K-12 and *S*. *flexneri* upon OAg inactivation. **A)** Western immunoblots of ECA of whole bacterial lysis samples from indicated bacterial strains. Detection of periplasmic marker SurA was used as a loading control. The status of OAg and ECA biogenesis for each strain is marked as OFF and ON. ECA detection levels were normalised against SurA and the ratio of ECA to SurA band intensities in MG1655 were defined as 100% to normalise all data. **B)** Surface ECA immunodetection via Epifluorescence microscopy. Scale bar shown as 5 μm. **C)** Quantification of ECA stained fluorescence intensity of whole bacteria, mean fluorescence intensity across each bacteria was used to perform quantification, n = 40. **D**) Quantification of ECA surface stained bacteria in the population of indicated bacterial strains quantified from three independent micrographs, at least 200 bacteria were counted per micrograph.

OAg in both WbbL-restored *E*. *coli* K-12 and *S*. *flexneri* initiates with GlcNAc, yet showed different degrees of cell viability due to UndP sequestration upon *wzyB* disruption (Fig 2C&3C). This prompted us to examine ECA production in both strains. We first confirmed that disruptions of both *wecG* and *wzyE* abolished the detection of ECA in both MG1655 and MG1655-S with anti-ECA antibodies, showing that the antibody is specific. Consistent with our above-proposed model, *E*. *coli* K-12 lacking OAg (MG1655) produced a high level of ECA in comparison to its OAg restored strain MG1655-S (Fig 5A). In contrast, in *S*. *flexneri* strain 2457T, inactivation of OAg biosynthesis by disruption of *rmlD*, involved in the synthesis of the precursor for the second sugar, L-Rhamnose within the OAg RU, only marginally increased the ECA production level (Fig 5A). Consistent with a previous study done in *S*. *flexneri* [27], disruption of *wzyB* in MG1655-S was also found with a slight increase in ECA production (Fig 5A). Disruption of *waaL* in both MG1655 and MG1655-S retained ECA production detected by Western immunoblotting, albeit with decreased signal intensity (Fig 5A). This is consistent with a previous report demonstrating that disruption of *waaL* in multiple bacterial strains greatly reduced reactivity with anti-ECA antiserum in whole cell agglutination experiment [28].

ECA exists in three different forms, i.e. ECA_LPS_, ECA_PG_ on cell surface and ECAcyc in the periplasm [12]. The formation of ECA_LPS_ is dependent on the O-antigen ligase WaaL [29]. Given that it is not feasible to detect ECAcyc molecule by Western immunoblotting due to its low molecular weight (~2,513 Da) [30], our polyclonal anti-ECA antibodies hence recognises both ECA_PG_ and ECA_LPS_. Mature ECA is predominantly located on the bacterial cell surface, and we therefore examined ECA surface production in both *E*. *coli* and *S*. *flexneri*. Consistent with the literature that surface labelling of ECA does not work in bacterial strains with LPS capped with OAg [31], the production of OAg on the cell surface masks ECA detection by anti-ECA antibodies for both *E*. *coli* K-12 MG1655-S and *S*. *flexneri* 2457T (Fig 5B). In contrast, inactivation of OAg production unexpectedly revealed that surface ECA could only be detected on approximately 30% of the cells in the *S*. *flexneri* Δ*rmlD* population, in comparison to 95% for *E*. *coli* K-12, albeit with similar surface ECA levels detected in ECA-positive bacterial cell for both strains (Fig 5B–5D). These results in part may explain the different tolerance to *wzyB* disruption between *E*. *coli* K-12 and *S*. *flexneri* (Fig 2C&3C), in that the capacity to make ECA in the *S*. *flexneri* Δ*rmlD* cell population is limited (30%) in comparison to *E*. *coli* MG1655 cell population (95%), leading to inadequate redirection of UndPP-GlcNAc into making ECA when *wzyB* is disrupted for the majority of the cell population (~70%). This limited ECA production in *S*. *flexneri* when OAg is disrupted renders it more susceptible to UndP sequestration, and ultimately cell death when WzyB is disrupted.

**Fig 6:**
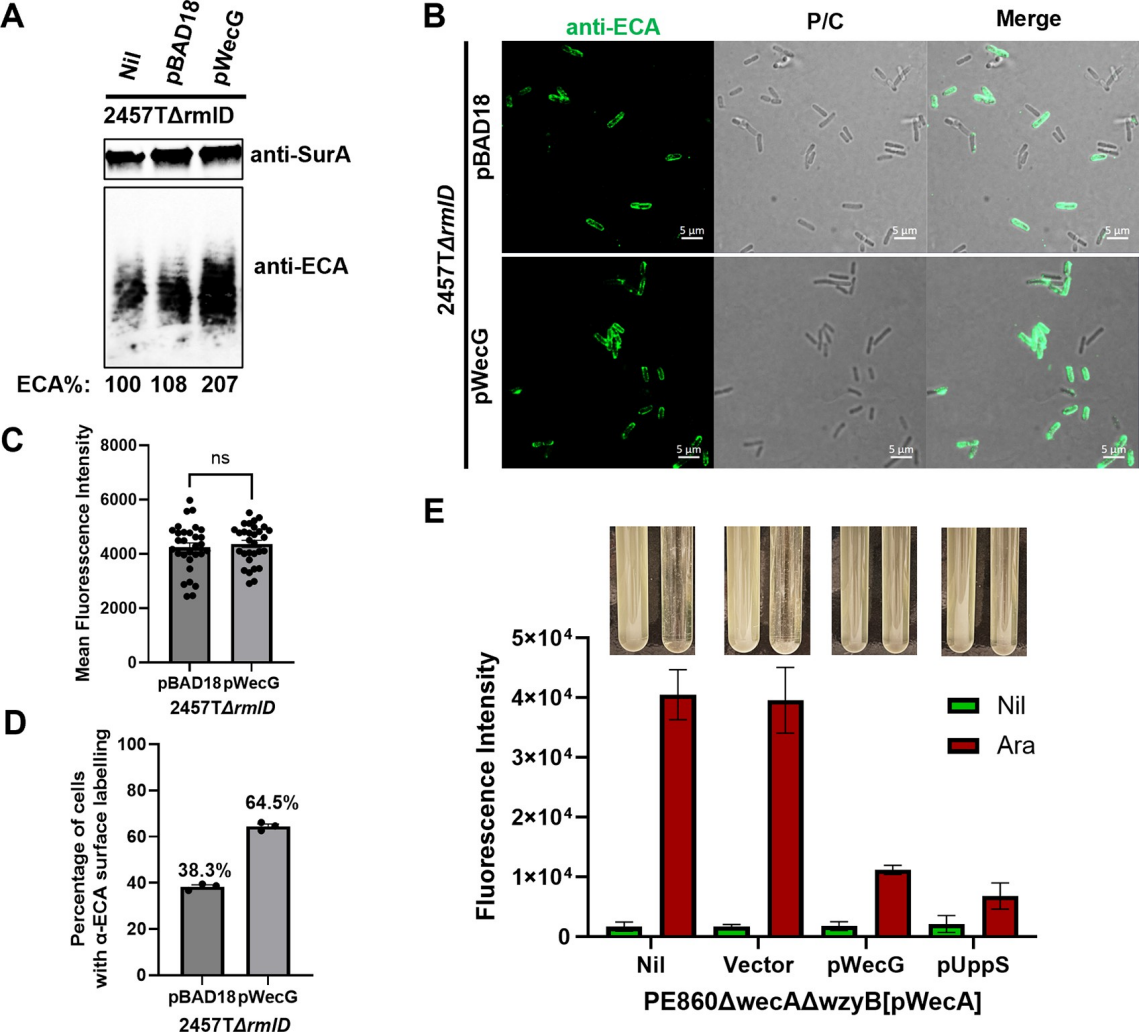
Expression of WecG increased ECA production and tolerance to UndP sequestration in *S*. *flexneri* Δ*wzyB* mutant. **A)** Western immunoblots of ECA in whole bacterial lysates from indicated bacterial strains. Detection of periplasmic marker SurA was used as a loading control. ECA detection levels were normalised against SurA and the ratio of ECA to SurA band intensities in 2457TΔ*rmlD* was defined as 100% to normalise all data. **B)** Surface ECA immunodetection via Epifluorescence microscopy. Scale bar shown as 5 μm. **C)** Quantification of ECA stained fluorescence intensity of whole bacteria, mean fluorescence intensity across each bacterial cell was used to perform quantification, n = 30. **D**) Quantification of ECA stained bacteria in the population of indicated bacterial strains quantified as percentages from three micrographs. At least 90 bacterial cells were counted per micrograph. **E)** Release of cellular DNA of PE860Δ*wecA*Δ*wzyB* with or without plasmids expressing WecG or UppS in culture supernatant upon pWecA induction detected by ethidium bromide (EtBr). Cell lysis was imaged as loss of turbidity in culture media.

To test this hypothesis, we ectopically over-expressed WecG in *S*. *flexneri* Δ*rmlD* and showed by Western immunoblotting that the ECA levels were increased by approximately 2-fold (Fig 6A). Interestingly, while surface ECA levels remained similar (Fig 6B–6C), the percentage of ECA-positive cells in *S*. *flexneri* Δ*rmlD* expressing WecG were increased to 64.5% in comparison to *S*. *flexneri* Δ*rmlD* carrying the vector control (Fig 6D). Intriguingly, expression of WecG greatly reduced the cell lysis of *S*. *flexneri*Δ*wecA*Δ*wzyB* mutant upon induction of WecA expression (Fig 6E). In addition, expression of UppS also rescued the lethality of the strain (Fig 6E). Taken together, these results suggest that limited ECA production in *S*. *flexneri* can account for its low tolerance to WzyB polymerase disruption and the lethality was due to the sequestration of UndP in UndPP-linked OAg intermediates.

## Discussion

Polysaccharide synthesis requires a cellular pool of universal lipid carrier UndP. Indeed, the production of OAg, while having no observable impact on growth, was shown to sequester a low level of UndP via UndPP-RU intermediates [22]. This was also supported here by the increased sensitivity of OAg-producing *E*. *col*i K-12 to bacitracin, an antibiotic that limits the availability of functional UndP. When the OAg synthesis pathway was disrupted beyond the committed steps, UndP was shown to be sequestered in UndPP-RU intermediates at a high level [22], severely affecting bacterial survival [13] due to deleterious effects on peptidoglycan synthesis [18]. Therefore, rapid assembly of polysaccharide RU on UndP carrier is favoured as it would lower the cellular demand for UndP at any given time.

### RU-synthesis controlled system reveals cell lethality outcomes

Disruption of OAg ligase *waaL* was shown previously to sequester high levels of UndPP-RU in OAg-producing bacterial strains [22]. However, an OAg-producing *E*. *coli* K-12 with *waaL* deletion had no growth impact here albeit was shown to induce a mild cell morphological defect previously [18] and was confirmed to lack suppressor mutations by whole genome sequencing. We have revealed here that in the OAg-producing *E*. *coli* K-12 strain MG1655-S, the polymerase WzyB which consolidates the RUs into polymeric forms and therefore rapidly releases UndPP in the absence of WaaL in the periplasm is crucial for cell viability. In a previous study [22], the level of C^14^ labelled OAg RUs on the isolated UndP pool was quantified in both *wzyB* and *waaL* mutants, with higher signal level of UndPP-linked OAg RU detected in the *wzyB* mutant. In the *wzyB* mutant, the stoichiometric relationship between OAg RU and UndP is 1:1, whereas it is (17–21):1 in the *waaL* mutant. Therefore, this suggests that a *wzyB* mutant sequestered a substantially higher level of UndP than a *waaL* mutant, suggesting that WzyB contributes to the rapid release of UndP from the OAg synthesis pathway. Supporting this notion, we have also found that in *S*. *flexneri*, deletion of *wzyB* is lethal. However, two *wzyB* mutants in *S*. *flexneri* have been reported previously. One of the only two reported *S*. *flexneri wzyB* mutants was acquired through Sf6c phage selection (*S*. *flexneri* Y OAg as its primary receptor) [32]. However, this mutant could not be fully complemented to confer resistance to Colicin E2 to WT level [33], a toxin whose entry is blocked by polymerised OAg [34]. Moreover, this mutant was later also used to derive a Δ*wzyB*Δ*waaL* double mutant in *S*. *flexneri* with no reported impact on growth [35], whereas it was shown here that deletion of both *wzyB* and *waaL* is lethal due to completely stalled UndPP-OAg intermediates. The other previously reported *S*. *flexneri wzyB* mutant [27] was acquired through direct allelic exchange mutagenesis after numerous attempts in the laboratory. The colony morphology of those putative mutants on the mutagenesis selection plate was similar as reported here, being overall small and heterogeneous in size. Like the other *S*. *flexneri wzyB* mutant that resulted from phage selection, this *wzyB* mutant could not be complemented to produce S-LPS to WT level [27]. Therefore, we believe that the two previously reported *wzyB* mutants likely contain suppressor mutations that have tuned down the committed step of OAg synthesis to reduce UndP sequestration for fitness, similar to suppressor mutations in *rml* genes reported previously in an OAg-producing *E*. *coli* K-12, making OAg at a reduced level [23].

While in a previous study [18], it was demonstrated that OAg dead-end intermediates due to genetic interruptions in OAg synthesis steps including flippase WzxB and late glycosyltransferases WbbJ and WbbK resulted in cell morphological abnormalities including loss of cell shape with bulges around cell envelope. The cell morphological defects in these mutants were due to the undersupply of UndP in peptidoglycan synthesis (which was sequestered in disrupted OAg synthesis pathways) as overexpression of UppS or MurA rescued these phenotypes [18]. Here we also generated evidence that expressing UppS could rescue the lethal phenotypes due to disruptions of WzyB in both *S*. *flexneri* and *E*. *coli* K-12 MG1655-S with inactive ECA production. Similarly, over-expression of WecG, the committed glycosyltransferase for ECA RU assembly, also rescued the cell morphological defects in mutants with disruptions in OAg biosynthesis [18] and showed here to alleviate the cell lysis in *S*. *flexneri* Δ*wzyB* mutant, consistent with our model here that the shared substrate UndPP-GlcNAc could be redirected into ECA synthesis pathway when OAg synthesis pathway is disrupted to allow adequate UndP recycling. However, whether these mutant strains with direct genetic disruptions incur cell lysis during various growth phases was not characterised. We argue that such mutants are highly likely to be genetically unstable, and this is well supported by work carried out by Nikaido and colleagues in 1969 [20], where studies with disruptions of OAg late glycosyltransferase made strains genetically unstable with unstable phenotypes, and the authors also reasoned that such mutation with UndP sequestration could result in additional peptidoglycan synthesis issue thus select for suppressor mutations that allow cell survival (revertant) with various phenotypes. Therefore, we avoided direct genetic disruptions of OAg synthesis pathways in MG1655-S as it may inevitably involve secondary selection of suppressor mutations to allow cell survival, producing phenotypes that may complicate the interpretation of results.

Together, these results caution future investigations on the impacts of potential UndP sequestration when disrupting genes responsible for polysaccharide synthesis, highlighting the necessity of conducting genetic studies on these genes using an expression controlled system at initial transferase or committed-step glycosyltransferase [13].

### Cell death requires high level of UndP sequestration

In an OAg production-controlled system (through tightly controlled expression of WbbL) employed in this work, we showed that the accumulations of OAg dead-end intermediates in OAg-producing *E*. *coli* K-12 Δ*waaL* and Δ*wzxB* mutants although were all documented with cell shape changes previously [18], only deletions of *wzxB* or *wzyB*/*waaL* in the OAg-producing *E*. *coli* K-12 caused cell lethality, which for the first time further distinguished the outcomes of UndP sequestration at different levels. Single deletions of *wzyB* or *waaL* was shown previously to accumulate UndPP-OAg intermediates [22], however only when disrupted together is the cell growth completely stalled. This is because UndPP-OAg-1RU is a common substrate competed by both WzyB and WaaL [11]. Under normal conditions, WaaL incorporates approximately 11% OAg-1RU into lipid A-core oligosaccharide [36]. However, in the absence of WzyB, WaaL ligates substantially more OAg-1RU onto lipid-A core oligosaccharide to form semi-rough LPS (SR-LPS, Fig 5A), thereby releasing adequate UndPP for recycling. In contrast, when both OAg ligase WaaL and polymerase WzyB are absent, the UndP employed in the common substrate UndPP-OAg-1RU would be sequestered fully, completely blocking the recycling path in the OAg synthesis pathway. This blockage results in lethality, consistent with the rationale for the cell death observed in *wzxB* deletion mutants.

Different cell death outcomes were previously documented in the ECA biosynthesis pathway, where only the gene deletion of *wzxE* caused lethality [25]. In two previous reports, disruptions of *rmlA* (in ECA gene cluster) [37] and *wecF* [38] that would accumulate intermediates, UndPP-GlcNAc-ManNAc, were successfully constructed, implying that such accumulation is not lethal. However, it is worth mentioning that the *rmlA* deletion could be in part functionally complemented by *rmlA* from the OAg gene cluster. This is supported in their work where a traceable amount mature ECA can still be detected [37], in which case, the ECA intermediate is not completely stalled to explain its viability. The *wecF* mutant was generated through direct transposon insertion [38] and whether it harbours secondary mutations is unclear. Nevertheless, the level of UndP sequestration in the situation where the incomplete RU is the dead-end intermediate (e.g. UndP-GlcNAc-ManNAc in a *wecF* deletion mutant) is predicted to be less than in the situation where the complete RU is the dead-end intermediate (e.g. UndP-GlcNAc-ManNAc- Fuc4NAc in a *wzxE* deletion mutant). This is because in the *wzxE* deletion mutants, UndP may be requested in both incomplete and complete ECA-RU dead-ends. This is clearly demonstrated in a previous report [39] where the Δ*wzxE* mutant sequestered the highest level of UndP in comparison to all disruptions in preceding steps of ECA synthesis. Therefore, it is likely that a UndP sequestration threshold exists to drastically impair peptidoglycan synthesis to induce cell death phenotype.

We showed here for the OAg synthesis pathway in MG1655-S, a Δ*wbbJ* deletion in the absence of ECA production caused cell death. We reasoned that the intermediates accumulated in a Δ*wbbJ* mutant would also completely stall the UndP sequestered in the disrupted pathway. This is consistent with previous studies showing that disruptions in late glycosyltransferases in OAg synthesis pathways in *Salmonella enterica* (whose OAg initiates with galactose rather than GlcNAc, therefore preventing redirection of the common substrate into the ECA pathway when OAg synthesis is disrupted) resulted in the accumulation of 10-fold more UndPP-linked intermediates [40], leading in cell death [41]. Therefore, the lack of redirection of the common substrate UndPP-GlcNAc into ECA synthesis pathway could result in low tolerance towards disruptions in OAg synthesis. In contrast, the accumulation of UndPP-GlcNAc-ManNAc intermediates in the two mutants (Δ*rmlA* and Δ*wecF*) mentioned above requires efficient supply of its donor substrate UDP-ManNAc. The substrate UDP-ManNAc was reported previously to be also utilised in the synthesis of a novel glycan for phage N4 infection [42]. This indicates an additional level of redirection of the common substrate UDP-ManNAc away from forming the dead-end intermediate UndP-GlcNAc-ManNAc, thus alleviate the severity of sequestration. Aside from ECA_LPS_, ECA polymers are also diverged to give ECA_cyc_ and ECA_PG._ While it may be tempting to correlate our phenotype to the intricate fate of ECA substrate, this would be overly speculative as the biosynthetic detail of the non-LPS forms remained unclear. Nevertheless, these data collectively support our model that common substrate could be redirected into intact synthesis pathways to limits the accumulations in the disrupted polysaccharide synthesis pathway.

### Increased ECA_LPS_ may in part account for the increased ECA production when OAg synthesis is disrupted

Contrasting to the essentiality of *wzyB* reported here in *S*. *flexneri*, a *wzyB* mutant was found to have no impact on growth in an OAg-producing *E*. *coli* K-12, and was reported previously with no suppressor mutations [23], hence being genetically stable. Both OAg of *S*. *flexneri* and *E*. *coli* K-12 are initiated with GlcNAc, which is the same initiating sugar for ECA. We generated evidence suggesting that *E*. *coli* K-12 redirects its UndPP-GlcNAc into ECA synthesis when OAg synthesis is disrupted, thereby alleviating the UndP sequestration stress. This was strongly supported with further experiments where inactivation of ECA synthesis rendered *wzyB* essential in OAg producing *E*. *coli* K-12. In contrast, we found that disruptions of OAg synthesis in *S*. *flexneri* only marginally increased overall ECA production, suggesting that it was unable to redirect adequate UndPP-GlcNAc from the disrupted OAg synthesis pathways, resulting in cell death due to UndP sequestration.

Interestingly, ECA was detected in very low levels in both MG1655Δ*waaL* and MG1655-SΔ*waaL* mutants through Western immunoblotting in comparison to MG1655 and MG1655-S. Disruption of *waaL* would abolish formation of ECA_LPS_, redirecting UndPP-ECA to the synthesis of other forms of ECA including ECA_cyc_. Given that it is not feasible to detect ECA_cyc_ by Western immunoblotting due to its low molecular weight, the production of ECA_cyc_ is unknown when *waaL* is disrupted. Therefore, it is possible that ECA production in MG1655Δ*waaL* and MG1655-SΔ*waaL* mutants could be severely underestimated. The detailed analysis of ECA_cyc_ is beyond the scope of this work as it is WzzE-dependent [30], and would warrant a separate study with new mutant combinations. Nevertheless, we could detect an increase in ECA production by Western immunoblotting in MG1655-SΔ*wzyB* in comparison to MG1655-S. These results therefore support our model that production of ECA is increased when OAg biosynthesis is disrupted.

ECA_LPS_ was known to be present in *E*. *coli* R1, R2, R4 and K-12 LPS core type, while not in R3 core type [31,43]. *S*. *flexneri* has LPS R3 core type, a non-permissive core type to form ECA_LPS_. Hence it is likely that the surface ECA stained in *S*. *flexneri* Δ*rmlD* mutant is the only other surface form, ECA_PG_. The result therefore is tempting to suggest that *S*. *flexneri* has limitations in producing ECA_LPS_ due to baring a non-permissive LPS core type, thereby having low capacity in redirecting UndPP-GlcNAc into making more ECA. However, although there is lack of characterisation of ECA forms in *S*. *flexneri* to confirm the absence of ECA_LPS_, ECA_LPS_ was reported to only exist in small amount (less than 5%) in rough LPS *E*. *coli* with different core types [31] and was proposed to be a by-product when OAg synthesis is inactive [43]. Indeed, silver staining of MG1655 LPS sample detected no ECA substituted LPS, albeit the sensitivity of the staining is at ng range [44]. Therefore, it is likely that the limitation of ECA production is not entirely due to the potential inability in ECA_LPS_ synthesis in *S*. *flexneri*. Supporting to this notion, we have also shown that overexpression of WecG in *S*. *flexneri*Δ*rmlD* increased both the ECA detection in Western immunoblotting, and the percentage of cells stained with ECA, and rescued the lethality in *S*. *flexneri*Δ*wzyB* mutant. Regardless of different forms of ECA may be detected by our ECA antibodies, the results presented here support our model in that increased ECA production leads to increased tolerance to the disruptions in OAg synthesis pathways.

### ECA is distributed heterogeneously in S. flexneri cell population

Interestingly, we unexpectedly found only a limited *S*. *flexneri* cell population (30%) decorated their cell surface with ECA upon disruptions of OAg synthesis. Since all bacterial cells were grown from a single bacterial colony for all experimental repeats, it is tempting to suggest that the surface ECA decoration in *S*. *flexneri* may be phase variable. This is the first example of surface ECA being heterogeneously displayed on the surface of *S*. *flexneri* cells in the population, but it remains unclear what mechanism underpins this. Since polysaccharide synthesis including the ECA synthesis pathway, lacks a feedback control mechanism, whereby disruptions in late steps of biosynthesis cause UndP sequestration leading to cell death [39], it is therefore likely to be regulated at the committed biosynthesis step, i.e. the committed glycosyltransferase WecG and enzymes (WecB and WecC) responsible for the synthesis of its nucleotide sugar substrate [42]. However, through sequence analysis between *S*. *flexneri* and *E*. *coli*, we were unable to identify any genetic regions that would potentially account for regulatory differences. Nevertheless, it may also be regulated through an additional molecular mechanism that is currently not known.

### Disruption of Wzy polymerases induces lethal phenotypes in polysaccharide biosynthesis pathways

Wzy polysaccharide polymerases for ECA in *E*. *coli* K-12 [26,45] was also reported to be essential. However, ECA polymerase *wzyE* was found not essential in uropathogenic *E*. *coli* OAg-producing strains [2,26], and here with an OAg restored *E*. *coli* K-12, highlighting the redirection of the common substrate UndPP-GlcNAc between ECA and OAg synthesis pathways as a mechanism to mitigate UndP sequestration stress. In addition, *wzyE* was found also essential in OAg producing *Salmonella* Typhimurium strains [26]. This provides further supporting evidence, because the OAg for *Salmonella* Typhimurium initiates with galactose [46], different to the ECA initiating sugar GlcNAc, and is thereby unable to redirect UndP-GlcNAc into synthesis of OAg.

Although numerous groups have characterised different enteric bacteria with saturated transposon insertional mutant libraries, we argue that the results may not reflect the gene essentiality accurately for the polysaccharide biosynthesis pathway. This is because to retain the mutant that may result in slow-growing phenotype, the mutant library may only be allowed for growing to early exponential phase, while that the cell lysis due to disruptions in polysaccharide synthesis genes may not occur immediately. This is evident in our results where the disruption of polysaccharide genes at late steps only starting to show cell lysis at mid-exponential phase at approx. OD_600_ of 0.4. Therefore, this growth-phase dependent lysis may not be well-captured in those studies with exhaustive mutagenesis. In particular, disruption of *wzxE* was shown here and previously [25] to be lethal in MG1655, while was shown not essential in other studies in mutant libraries [47,48]. Another complication in studies through direct inactivation of polysaccharide genes was unintentional selection of potential secondary suppressor mutations allowing survival of the mutant with targeted mutation in polysaccharide genes, similar to the selection of a *S*. *flexneri* Δ*wzyB* duplication suppressor mutation demonstrated in this study.

It is worth noting that cell death may not be the only outcome when late biosynthesis steps are disrupted for other polysaccharides. In our study, the mechanism of cell death was specifically attributed to the sequestration of UndP, which limits its availability for the peptidoglycan biosynthesis pathway. However, several alternative mechanisms can mitigate cell death when UndP is sequestered in the biosynthesis pathway: 1) In polysaccharide pathways where production is generally less active during the exponential growth phase and more active during the stationary phase—when peptidoglycan biosynthesis is also less active—or at lower temperatures, which slow cell growth and reduce the demand for UndP in peptidoglycan biosynthesis, as seen in exopolysaccharide biosynthesis [49]; 2) In cells that carry genes with redundant functions, such as the *rml* genes present in both the ECA and OAg pathways; and 3) In certain bacterial species, where the availability of UndP may be higher. However, cell death outcomes have been widely observed for other polysaccharides, including capsule polysaccharide [21], exopolysaccharide succinoglycan [50] and protein O-linked glycan [51], when late biosynthesis steps are disrupted, as previously summarised [52]. Therefore, the essentiality of polysaccharide genes must be carefully studied within a polysaccharide synthesis-controlled system to avoid the complications mentioned above.

### Sharing the initial substrate with ECA biosynthesis pathway provides benefit in OAg diversification adaptation process

The diversification of the OAg RU structure presents significant challenges due to the high specificity of the enzymes involved in RU assembly and processing, such as glycosyltransferases, flippases, and polymerases. When OAg structure is modified at the cytoplasmic leaflet of the IM, these existing enzymes may not recognise the altered UndPP-RU, leading to stalled synthesis. This disruption in the pathway can result in UndP sequestration and ultimately cause cell lysis, which potentially imposes evolutionary constraints for further diversification processes. Interestingly, in contrast to *E*. *coli*, the diversification of *S*. *flexneri* OAg structures is primarily restricted to modifications introduced by bacterial phage elements, which encode enzymes modifying OAg structures on the periplasmic face of the inner membrane [53]. This approach allows *S*. *flexneri* to bypass the specificity of the enzymes responsible for OAg RU biosynthesis. Our data suggest that, in addition to the pathogenic importance of *S*. *flexneri* OAg structures, a reduced capacity for ECA synthesis could pose an evolutionary constraint to its OAg diversification. Specifically, this limitation could impede the ability of *S*. *flexneri* to adapt its OAg structures efficiently through genetic recombination to acquire a new glycosyltransferase or other alterations leading to the evolution of a novel OAg structure, in that the limited tolerance to disrupted flows in OAg synthesis would quickly eliminate the recombinants before the acquisition of adaptive changes to evolve glycosyltransferase and flippase compatibility. We argue this may explain the restricted OAg structural diversity in *S*. *flexneri* compared to other *E*. *coli* lineages.

**Fig 7:**
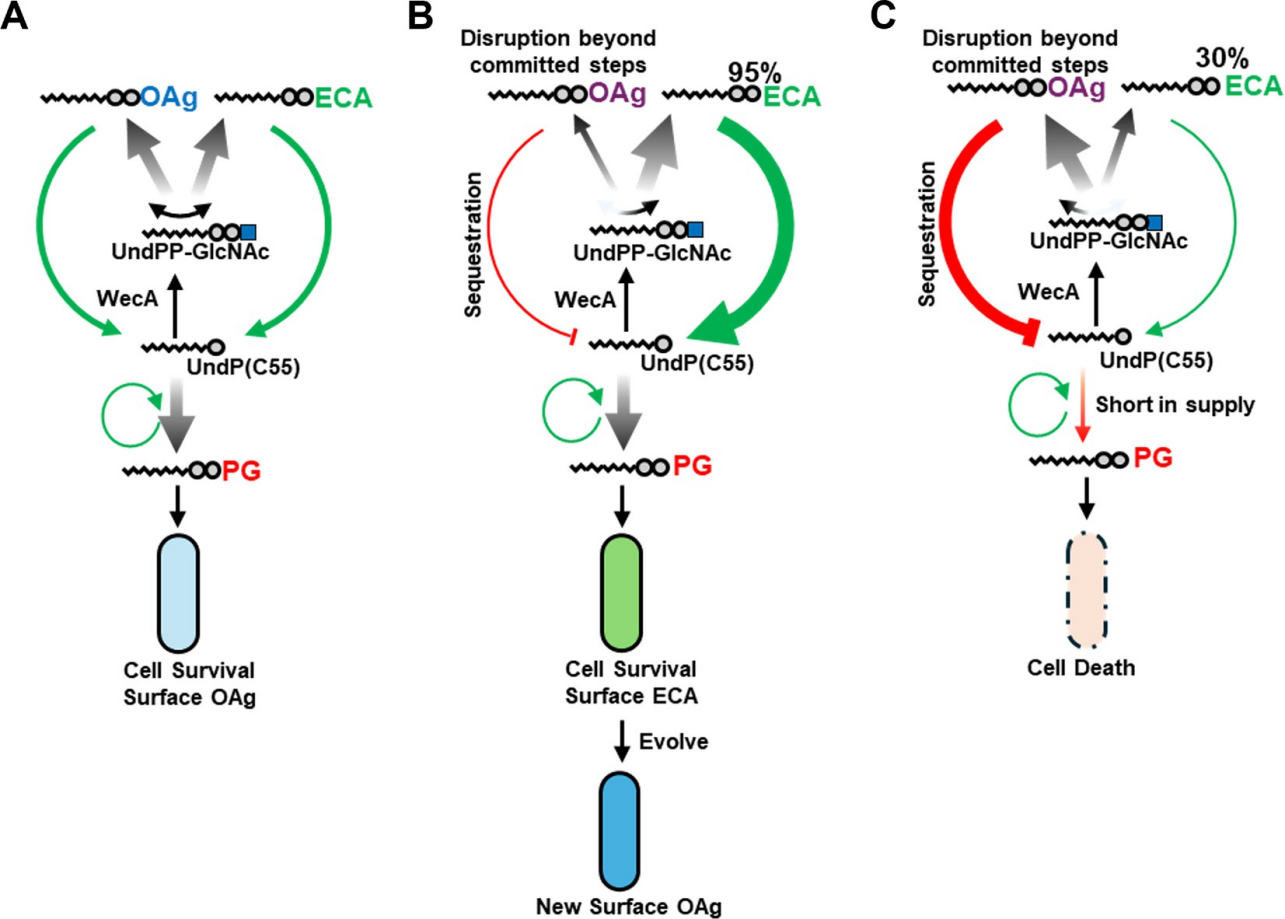
A model of the buffering mechanism that redirects UndP into ECA biosynthesis to maintain rapid UndP recycling during OAg pathway stalling or disruption. **A)** OAg and ECA RU assembly shares the initial substrate UndPP-GlcNAc which is catalysed by initial transferase WecA to engage the lipid carrier UndP. The availability of UndP to peptidoglycan biosynthesis is crucial for cell survival. When OAg biosynthesis is disrupted beyond the committed steps, UndPP-RU intermediates accumulate on the IM, locking UndP into the OAg biosynthesis pathway. **B)** This can be mitigated by redirecting UndPP-GlcNAc into a robust ECA biosynthesis pathway to maintain rapid UndP recycling rates. This cell survival supports further diversification of the new OAg. **C)** when the ECA pathway has limited biosynthesis capacity, disruptions in OAg biosynthesis may result in cell death halting further evolution process of OAg.

Most *E*. *coli*, *Shigella* and other Enterobacteriaceae have their OAg RU initiates with GlcNAc (or subsequently modified to from GlcNAc to GalNAc by epimerase on the lipid carrier in a reversible manner [9]) as their first sugar by the initial transferase WecA [6] encoded in the ECA gene cluster, thereby sharing the first RU intermediate UndPP-GlcNAc with ECA RU synthesis. We propose a model in which sharing the initial sugar, GlcNAc, between OAg and ECA RUs allows for the redirection of UndPP-GlcNAc when one pathway encounters stalling or disruptions. This mechanism helps mitigate the stress caused by UndP sequestration (Fig 7A). Therefore, ECA synthesis with a high level of production would enhance the cell’s tolerance to UndP sequestration caused by disruptions in OAg synthesis. This increased production allows the cell to temporarily decorate its surface with ECA, which is crucial for maintaining cell survival while enabling further diversification of the new OAg (Fig 7B). Indeed, *E*. *coli* O14 (ECA) reference strain Su4411-41 decorating cell surface with ECA was found with OAg gene cluster deleted via homologous recombination [54]. The same situation was found with other rough LPS *E*. *coli* strains devoid of OAg, where these strains decorate their surface with O14 (ECA) [31,43]. In contrast, limited ECA production results in low tolerance to disruptions in OAg biosynthesis, leading to cell death and potentially imposing constraints on the evolution process (Fig 7C).

## Conclusion

Our work is of great importance as only by revealing the death outcomes of these disruptions in late steps of OAg biogenesis, could we understand the significant hurdles of which OAg diversification may have to face, i. e. disruptions in late steps of OAg biosynthesis may cause UndP sequestration leading to cell death. This was completely overlooked due to the lack of characterisation of growth phenotypes of mutant strains with disruptions in late steps of polysaccharide synthesis. Our work here thus pointed out once again the importance of using an initial/committed-step glycosyltransferase-controlled system studying OAg biosynthesis that involved in genetic disruptions of subsequent assembly steps to report phenotype for interpretation based on genetically stable mutants strains, and provided with further evidence that robust ECA synthesis could help in redirection of the common substrate UndPP-GlcNAc of ECA and OAg, thereby alleviating the degree of UndP sequestration and allowing continuation of further genetic adaptation.

## Methods and materials

### Bacterial strains and plasmids

Bacterial strains and plasmids used in this work are listed in Table 1. Single colonies grown on Lysogeny Broth (LB)-Lennox [55] agar plates were picked and grown overnight in LB at 37°C for all experiments. Where appropriate, LB media were supplemented with ampicillin (Amp, 100 μg/mL), kanamycin (Kan, 50 μg/mL), chloramphenicol (Chl, 25 μg/mL), anhydrotetracycline (AhTet 50 ng/mL), or arabinose (Ara, 10 mM).

**Table 1:** Strains, plasmids, and oligonucleotides.

**Bacterial strains**		
**Strains**	**Description**	**Source**
MG1655	Wild-type *E*. *coli* K-12 MG1655	Lab stock
MG1655Δ*wecG*	MG1655Δ*wecG*::*neo*	This work
MG1655-S	MG1655 with IS5I removed in *wbbL*	[23]
MG1655-SΔ*wecA*	MG1655-SΔ*wecA*::*frt*	[23]
MG1655-SΔ*wzyE*	MG1655-SΔ*wecA*::*neo*	This work
MG1655-SΔ*wecG*	MG1655-SΔ*wecG*::*neo*	This work
MG1655-SΔ*wecG*	MG1655-SΔ*wecG*::*frt*	This work
MG1655Δ*wecG*Δ*wzxE*	MG1655Δ*wecG*Δ*wzxE*::*neo*	This work
MG1655-SΔ*wecA*Δ*wzyE*	MG1655-SΔ*wecA*::*frt*Δ*wzyE*::*neo*	This work
MG1655-SΔ*wecA*Δ*wecG*	MG1655-SΔ*wecA*::*frt*Δ*wecG*::*neo*	This work
MG1655-SΔ*wecA*Δ*wecG*Δ*wzyB*	MG1655-SΔ*wecA*::*frt*Δ*wecG*::*neo*Δ*wzyB*::*cat*	This work
MG1655-SΔ*waaL*	MG1655-SΔ*waaL*::*frt*	[23]
MG1655Δ*waaL*	MG1655Δ*waaL*::*frt*	[23]
MG1655-SΔ*wzyB*	MG1655-SΔ*wzyB*::*cat*	[23]
MG1655-SΔ*waaL*Δ*wzyB*	MG1655-SΔ*waaL*::*frt*Δ*wzyB*::*neo*	This work
MG1655-SΔ*waaL*Δ*wzyB*Δ*wbbJ*	MG1655-SΔ*waaL*Δ*wzyB*::*neo*Δ*wbbJ*::*cat*	This work
PE860	*Shigella flexneri* Y serotype	[27]
PE860ΔwzyB*	PE860ΔwzyB*::*neo*, *contains WzyB duplication mutation*	This work
PE860Δ*wecA*	PE860Δ*wecA*::*frt*	[13]
PE860Δ*wecA*Δ*wzyB*	PE860Δ*wecA*::*frt* Δ*wzyB*::*neo*	This work
2457T	*Shigella flexneri* 2a serotype	[56]
2457TΔ*rmlD*	2457TΔ*rmlD*::*neo*	[57]
**Plasmids**		
**Plasmids**	**Description**	**Source**
pBAD322G	Cloning plasmid, arabinose promoter, Gent^R^	[58]
pKD46	Temperature sensitive plasmid expressing Red proteins, Amp^R^	[59]
pCP20	Plasmid expressing FLP flippase, Amp^R^	[59]
pKD4	Plasmid carrying FRT flanked kanamycin resistant cassette, Amp^R^, Kan^R^	[59]
pKD3	Plasmid carrying FRT flanked kanamycin resistant cassette, Amp^R^, Chl^R^	[59]
pWQ572	Tetracycline inducible promoter, Chl^R^	[60]
pWbbL	*wbbL* CDS cloned from WG1 into pWQ572	[61]
pWecA	*wecA* CDS cloned from MG1655 into pBAD322G	[13]
pWecG	*wecG* CDS cloned into pBAD18cm	[62,63]
pUppS	*uppS* CDS cloned into pBAD18cm or pWSK30	[62,63]
**Oligos**		
**Description**	**Sequence**	
*wecA* KO F	TCGGTTTACGCAGGGATTTGCTTCACGTTCGGAATTGTCGGTGTAGGCTGGAGCTGCTTC	
*wecA* KO R	CTGCGTTTTACGCGCTTAATAAAGCGAGCAACTTTCCAGGATGGGAATTAGCCATGGTCC	
*wzyE* KO F	TGTTTGTTGTCTGGCTGCTCTGCACGCTGTTTATTGCCACGCTGACCTGGATGGGAATTAGCCATGGTCC	
*wzyE* KO R	TTTGTACGTTTATGAATGAGTCCGGCGCTTTCAAAAAGCCAGTACAACAGGTGTAGGCTGGAGCTGCTTC	
*wzyE check F*	AATTGGTACCATGAGTCTGCTGCAATTCAG	
*wzyE check R*	AATTGGATCCTTATCCTTCAACCTGCGTCC	
*wecG* KO F	ACACCACGGCACCAACCTATACGCTGCGTGGCTTACAGTTGATTGGTTGGATGGGAATTAGCCATGGTCC	
*wecG* KO R	AGGTTGCCGGTGTAGTGCCAGCGTAAATAACGCAGCAAACGAAGCTGACGGTGTAGGCTGGAGCTGCTTC	
*K-12 wzyB KO F*	CGCTCTTTATCAAGTGAAAAATATAATGAGTACGGATTAAGTGTAGGCTGGAGCTGCTTC	
*K-12 wzyB KO R*	CGCGTCTAGAGAAATTTAAATCATTCAAAAAATACATTTTATGGGAATTAGCCATGGTCC	
*SF wzyB KO F*	TAAATAAAATTTTTATAACATTTTTATGTATTGAACTGATTATTGGTGGTATGGGAATTAGCCATGGTCC	
*SF wzyB KO R*	GCTCCAGAAGTGAGGTTATTACTAATTTGGATATTTTCTATAGAAAATACGTGTAGGCTGGAGCTGCTTC	
*SF wzyB Check F*	AATTGGTACCATGAATAATATAAATAAAATTTTTATAACATTTTTATGTATTGAACTG	
*SF wzyB Check R*	AATTGGATCCTTATTTTGCTCCAGAAGTGAGG	

### Bacterial mutagenesis via allelic exchange

Mutagenesis was performed as previously described [59] with modifications [64]. Bacterial strains with plasmid pKD46 were grown overnight in 10 mL LB at 30°C and diluted 1 in 100 into 10 mL LB. Lambda Red protein expression was induced with 50 mM L-arabinose when OD_600_ reached 0.3 and continued for 1 hour. Cells were then centrifuged (5,000 ◊g), washed with ice-cold water, and resuspended in 100 μL of 10% ice-cold glycerol for electroporation. The *cat* or *neo* gene was PCR-amplified from pKD3 or pKD4, respectively, using primers with 40–50 bp of homologous sequences (Table 1). The purified PCR product (1.5 μg) was introduced into electrocompetent cells by electroporation. After recovery in 3 mL LB at 37°C for 2 hours, cells were plated on LB agar with Chl or Kan and incubated at 37°C for 16 hours. Mutants were then confirmed by PCR screening.

### Bacitracin sensitivity assay

Bacterial survival spotting assays were conducted as previously described [23]. Overnight bacterial cultures were adjusted to an OD_600_ of 1.0, then serially diluted 10-fold to 10^−7^ in fresh LB media. A 4 μL aliquot of each dilution was spotted onto LB agar plates, with or without 1 mg/ml bacitracin (Sigma, B0125).

### Bacterial growth kinetic assay

Bacterial growth kinetics were recorded as described previously [23]. Overnight bacterial cultures were diluted 1:200 in fresh LB media, with or without 10 mM arabinose and/or 50 ng/mL anhydrotetracycline, in a 96-well plate. The plate was incubated at 37°C with aeration, and OD_600_ was measured every 10 minutes for 18 hours using a CLARIOstar plate reader (BMG, Australia).

### Bacterial cell lysis assay

To measure bacterial cell lysis, WecA and WbbL were induced in bacterial cells grown to an OD_600_ of 0.8–1 and incubated for an additional 30 minutes at 37°C. Cultures showing reduced turbidity due to cell lysis were imaged. The supernatants were then collected by centrifugation at 20,000 × g and mixed with 5 μg/mL Ethidium bromide (EtBr, BioRad). Fluorescence was measured using a CLARIOstar plate reader (BMG, Australia) with excitation at 525 nm and emission at 615 nm.

### LPS silver staining

For LPS silver staining, bacterial cells (10_9_) were harvested via centrifugation, and lysed in 50 μl of SDS sample buffer. Samples were then heated at 100°C for 10 min, then treated with 20 μg/ml proteinase K (NEB, #P8107S) overnight at 60°C for 18 hours. Samples (3–5 μl) were then separated by SDS-Tris/Glycine gel (BioRad, #4568095) electrophoresis and subjected to LPS silver staining as detailed previously [44].

### Western immunoblotting

For western immunoblotting of ECA, the above samples prepared for LPS silver staining were separated by SDS-Tris/Glycine gel (BioRad, #4568095) electrophoresis and subsequently transferred onto nitrocellulose membrane and detected with rabbit polyclonal anti-ECA antibodies [27]. For loading control, lysed whole cell bacterial samples without proteinase K treatment were separated by SDS-PAGE and subsequently immunoblotted with anti-SurA antibodies (gifted by Carol Gross, University of California). For comparisons of ECA detection between samples, densitometry analysis was performed by using Image Lab (Version 6.1.0, BioRad). Background adjusted band intensity volume of detected SurA signals were used to normalise the band intensity volume of detected ECA signals.

### Surface ECA immunostaining

For ECA surface labeling, bacteria (10^8^ cells) in mid-exponential phase were harvested by centrifugation (16,000 × g, 1 min), fixed with 3.7% (wt/vol) formaldehyde in PBS for 20 minutes at room temperature, and then washed with PBS. A 5 μL suspension of fixed bacteria was centrifuged onto coverslips precoated with 0.01% (wt/vol) poly-L-lysine (Sigma) in a 24-well tray (16,000 × g, 1 min). Coverslips were incubated sequentially with rabbit anti-ECA pAbs (1:100), followed by anti-rabbit Alexa Fluor 488 (Invitrogen, 1:100) in PBS with 10% (vol/vol) FBS (Gibco), with PBS washes in between. Coverslips were mounted with ProLong Diamond Antifade Mountant (Invitrogen) and imaged using a ZEISS Axio Vert.A1 microscope. The experiment was carried out twice, and the mean intensity of ECA signals across each whole bacterium and the percentage of ECA-positive bacteria was quantified using three micrographs in ZEISS ZEN blue (Version 3.6, Carl Zeiss).

## Supporting information

S1 Table Raw data for growth kinetics. Recorded values of optical density at 600 nm of bacterial cultures growing at 37°C.
(XLSX)
